# Trematode-Associated Renal Lesions in Stranded Humboldt Penguins (*Spheniscus humboldti*) Along the Chilean Coast

**DOI:** 10.3390/ani16060859

**Published:** 2026-03-10

**Authors:** Carlos A. Flores Olivares, Juan Pablo Ruíz Yañez, Gerardo Cerda, Sofía Marambio, Tomás Pino, Maximiliano Schultz, Pablo Oyarzún-Ruiz, Carlos Sandoval

**Affiliations:** 1 Laboratorio de Ciencias Veterinarias, Universidad del Alba, La Serena 1700000, Chile; jpruiz@udalba.cl; 2 Servicio Nacional de Pesca y Acuicultura, Coquimbo 17800000, Chile; gcerda@sernapesca.cl (G.C.); smarambio@sernapesca.cl (S.M.); 3 ONG, Conservación Humboldt, Coquimbo 17800000, Chile; conservacionhumboldt.ong@gmail.com (T.P.); maxvetschultz@gmail.com (M.S.); 4 Departamento de Microbiología, Facultad de Ciencias Biológicas, Universidad de Concepción, Concepción 4081407, Chile; poyarzun@udec.cl; 5 Veterinary Histopathology Center, Puerto Montt 548000, Chile; carlos.sandoval@vehice.com

**Keywords:** *Spheniscus humboldti*, kidney diseases, renicola

## Abstract

Humboldt penguins (*Spheniscus humboldti*) are classified as Vulnerable and listed in Appendix I of CITES, making the investigation of stranding and mortality causes essential for their conservation. This study describes the post-mortem findings of five Humboldt penguins stranded along the Chilean coast during 2025, focusing on renal and ureteral lesions associated with trematode infection. Gross examination revealed multifocal to coalescing renal lesions, including intrapelvic white purulent material and marked thickening of the ureteral walls. Histopathological analysis demonstrated moderate to severe renal and ureteral inflammation, characterized by intratubular and intraureteral trematodes associated with tubular degeneration, interstitial inflammation and fibrosis, vascular alterations, occasional glomerular changes, and severe ureteritis. Morphological and morphometric analyses of adult parasites obtained from histological renal sections confirmed the presence of trematodes. On clinical evaluation, stranded animals presented with lethargy, no evidence of external trauma, and body condition ranging from normal to emaciated. These findings confirm the presence of trematode-associated nephropathy and ureteropathy in Humboldt penguins. To our knowledge, this is the first report worldwide linking renal and ureteral parasitosis to disease in this species. Further molecular analyses are required to achieve definitive etiological identification and to support the recognition of this condition as an emerging disease relevant to conservation strategies for Humboldt penguins.

## Simple Summary

Humboldt penguins are a vulnerable species whose population is continuously declining along the Chilean coast. Although infectious diseases may play a role in penguin mortality and stranding events, renal diseases caused by parasites have not been previously reported in this species. In this study, we describe renal and ureteral inflammation associated with trematode parasites in five stranded Humboldt penguins. While clinical examinations of penguins that were stranded alive did not show obvious signs of renal disease, post-mortem examinations revealed severe lesions affecting the kidneys and ureters. These findings suggest that parasitic renal infections may represent an emerging health threat for Humboldt penguins and could contribute to stranding and mortality. Improved recognition of this condition may support better diagnosis, rehabilitation, and conservation strategies for this vulnerable species.

## 1. Introduction

The Humboldt penguin (*Spheniscus humboldti*), a species within the order Sphenisciformes and the family Spheniscidae, is distributed along the Pacific coasts of Peru and Chile, ranging from La Foca Island (05°12′ S, 81°12′ W) to Guafo Island (43°32′ S, 74°42′ W) [1]. The largest breeding colonies are in Chile [2,3]. The diet of this species consists primarily of fish, including anchovy (*Engraulis ringens*), Araucanian herring (*Strangomera bentincki*), silverside (*Odontesthes regia*), common hake (*Merluccius gayi*), Inca scad (*Trachurus murphyi*), South American pilchard (*Sardinops sagax*), and garfish (*Scomberesox saurus scombroides*), as well as cephalopods and crustaceans, mainly malacostracans and isopods [4].

Globally, the Humboldt penguin is classified as Vulnerable, with a declining population estimated at approximately 23,800 mature individuals, according to the International Union for Conservation of Nature (IUCN) [1]. Approximately 80 breeding colonies have been reported throughout its distribution range, including 42 in Peru and 38 in Chile [5]. The species is also listed in Appendix I of the Convention on International Trade in Endangered Species of Wild Fauna and Flora (CITES). In Chile, the Humboldt penguin is likewise classified as Vulnerable, and its Recovery, Conservation, and Management Plan (RECOGE) identifies seven major threats: invasive exotic species, fisheries interactions, anthropogenic disturbance, harmful productive practices and civil works, human consumption, free-roaming dogs, and emerging diseases. These emerging diseases include ocular pathologies and highly pathogenic avian influenza [6] (Decreto N°1, 25 July 2024, Ministerio del Medio Ambiente, Chile).

Despite the recognized impact of infectious diseases on this species, trematode-associated renal and ureteral inflammation has not previously been reported in Humboldt penguins. Our research group frequently observed this condition during necropsies performed between July and November 2025. Therefore, this study presents the first report of trematode infection associated with severe tubular, interstitial, and ureteral lesions, as well as marked renal fibrosis, in stranded Humboldt penguins along the Chilean coast during 2025.

## 2. Materials and Methods

### 2.1. Stranding Records

The study included five Humboldt penguins (*Spheniscus humboldti*) that stranded alive or dead along the Chilean coast in 2025. All animals were donated under official authorization issued by the National Fisheries and Aquaculture Service (SERNAPESCA), Coquimbo, Chile. Recorded data included individual identification number, stranding condition (alive or dead), age category, sex, body condition score [7], stranding date, date of death, and geographic location.

### 2.2. Necropsy and Histopathology

Necropsies were performed at the Laboratory of Veterinary Sciences Research (LiCiVet), Universidad del Alba, La Serena campus, Chile. A complete gross examination of all organs was conducted. Multiple representative tissue samples from different organs were collected and fixed in 10% neutral buffered formalin (DiaPath, Martinengo, Italy). Formalin-fixed tissues were submitted to the Veterinary Histopathology Center (VeHiCe), Puerto Montt, Chile, for routine histological processing. Tissue sections were stained with hematoxylin and eosin (H&E), periodic acid–Schiff (PAS), and Masson’s trichrome. Histological slides were subsequently digitized using a Motic Easy Scan system at magnifications of up to 80×.

### 2.3. Analysis and Identification of Parasitic Structures

Parasites were morphologically analyzed, and their distinct anatomical structures were identified [8,9,10]. The developmental stage of the trematodes was evaluated based on morphological criteria and the presence of mature reproductive structures, including a uterus filled with eggs [10]. Morphological and morphometric analyses were performed on adult parasites from histologic sections, including measurements of body length and width. In addition, eggs were quantified and measured within the uterus of individual adult trematodes. A descriptive statistical analysis was conducted, including mean values, standard deviations, and minimum and maximum measurements for both adult parasites and eggs, as well as egg counts per individual parasite [10,11].

## 3. Results

### 3.1. Stranding Records

Of the five Humboldt penguins analyzed, one stranded dead and four stranded alive between July and November 2025. Both adult and juvenile individuals were included, comprising males and females, with body condition scores ranging from emaciated to normal. The four live-stranded penguins were transferred to the Humboldt Conservation Rehabilitation Center, where they died during rehabilitation within 0–14 days post-admission (Table 1).

### 3.2. Necropsy and Histopathology

**Figure 1:**
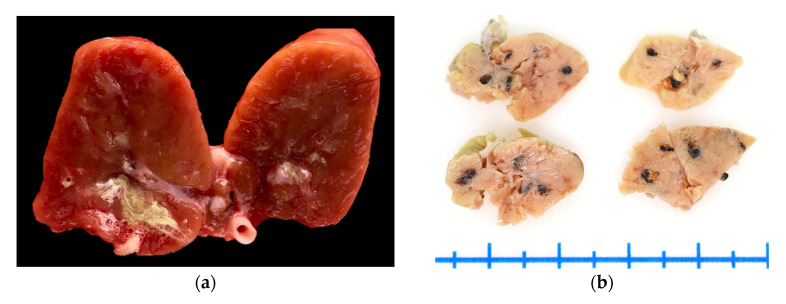
Kidneys of Humboldt penguins with trematode-associated lesions. (**a**) Animal ID 1: Fresh tissue showing purulent-appearing material within the renal pelvis. The ureter is thickened in the center of the image. (**b**) Animal ID 4: Four sections of tissue fixed in 10% buffered formalin. Darkened areas correspond to dilated renal tubules containing adult parasitic structures.

On gross examination, the kidneys (fresh tissue) from the animals exhibited marked vascular congestion with hyperemic vessels, white intratubular structures, and mucopurulent exudate, which were most evident in animal ID: 1 (Figure 1a). The renal pelvis was markedly distended and contained urate deposits; the ureters were dilated and showed pronounced wall thickening. During the selection of areas for microscopic analysis in animal ID: 4, rounded black areas measuring up to 0.2 cm in diameter were observed, with a multifocal to coalescing distribution within the renal parenchyma (Figure 1b). Animals ID: 2, 3, and 4 exhibited serous atrophy of fat, with up to 15 mL of free translucent fluid in the pericardial sac (hydropericardium).

**Figure 2:**
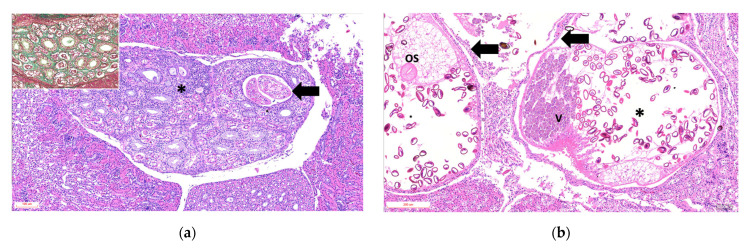
Photomicrographs of histopathological sections of kidneys from Humboldt penguins with trematode-associated lesions in collecting tubules (H&E). (**a**) Animal ID 1: Arrow indicates dilated renal tubules measuring 350 × 219 µm (length × width) containing two parasitic structures at stage 2 within the tubular lumen. Asterisk: Peritubular tissue with marked diffuse interstitial lymphoplasmacytic inflammation (H&E—100× magnification). Inset: Green staining highlights interstitial collagen deposition (Masson’s trichrome). (**b**) Renal tubules dilated up to 1123 × 767 µm. Black arrow indicates the epithelial wall surrounding adult parasitic structures. Asterisk highlights a markedly dilated uterus containing multiple oval to round eggs covered by a thick eosinophilic capsule (Opercule); miracidia were occasionally observed within the eggs (H&E—100× magnification). V: vitellaria; OS: oral sucker. Adjacent renal tissue shows areas of mononuclear inflammation, degeneration, and necrosis.

Histological analysis was performed on 5 penguins, and the findings are summarized in the Supplementary Table S1. The main tubular lesions were characterized by degeneration of the epithelial cells of the collecting tubules, cystic dilatations containing one or two parasitic structures (Figure 2a), and adult parasites with uteri containing variable numbers of eggs (Figure 2b). Tubules containing urate crystals surrounded by lymphoplasmacytic inflammation with heterophils were observed in 3 of 5 animals. The interstitium exhibited moderate to marked diffuse fibrosis, with bands of mature collagenous tissue between tubular components (Figure 2a, Inset). The vascular component was hyperemic and congested, with multiple hemorrhagic foci.

**Figure 3:**
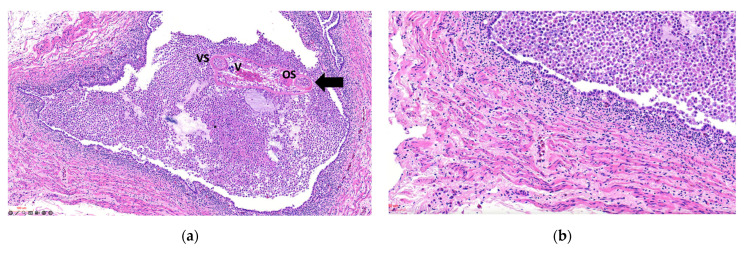
Photomicrographs of ureters from a Humboldt penguin with severe inflammation (Animal ID 2) affected by trematodes. (**a**) Dilated ureter measuring 1353 × 854 µm with epithelial wall showing areas of degeneration and necrosis. A trematode is present in the lumen, surrounded by abundant macrophages, cellular debris, heterophils, and granular eosinophilic material. VS: ventral sucker; OS: oral sucker; V: vitellaria (H&E—100× magnification). Black arrow: Adult parasite. (**b**) Smooth muscle tissue with hyper-eosinophilic, fragmented, and necrotic fibers infiltrated by lymphocytes, plasma cells, and heterophils (H&E—200× magnification).

Glomeruli occasionally showed expansion of the urinary space with areas of increased cellularity. The ureters exhibited luminal dilation, epithelial hyperplasia, epithelial cell sloughing, squamous metaplasia, and necrosis, with multiple intraluminal parasitic structures surrounded by abundant macrophages, cellular debris, and heterophils in all cases, varying in severity (Figure 3a). The smooth muscle tissue adjacent to the ureters showed variable inflammation, mainly degenerative and necrotic lesions of muscle fibers, with moderate mononuclear inflammation on a pale proteinaceous background and edema (Figure 3b). In the analyses performed, no evidence of other infectious agents was observed that could be associated with the inflammatory response in the kidneys and ureters.

### 3.3. Analysis and Identification of Parasitic Structures

**Figure 4:**
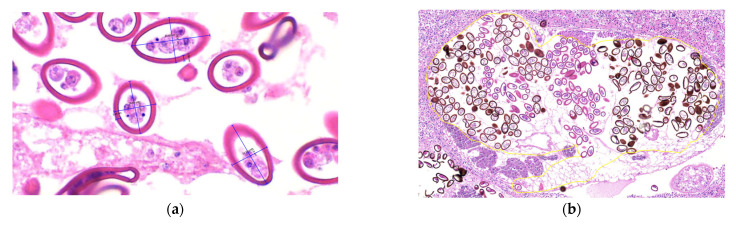
Morphometric measurements and egg count in samples from Animal ID 4. (**a**) Measurement of egg length, width, and capsular (opercular) thickness (µm) measured 45.9 ± 8.51 × 26.14 ± 3.55 µm (H&E—400× magnification). (**b**) Dilated renal tubule containing an adult trematode with numerous eggs within the uterus (outlined in yellow) (H&E—100× magnification).

Morphologically, the parasites exhibited an eosinophilic tegument with abundant tegumentary spines, a clearly identifiable digestive tract, and reproductive structures including testes, uterus, and vitellaria, as well as a muscular pharynx and both oral and ventral suckers. Based on these features, the parasites were classified as trematodes. Parasites were observed at different developmental stages. In stages 3, 4, and 5, eggs were identified within the uterus, with or without visible miracidia. Adult parasites (n = 20) measured 1175 ± 319.77 × 811.33 ± 303.70 µm (length × width; mean ± SD). Egg morphometric data are summarized in the Supplementary Table S2. Eggs within renal lesions (n = 583) measured 45.9 ± 8.51 × 26.14 ± 3.55 µm, with a capsular (opercular) thickness of 3.68 ± 0.60 µm (length × width × capsule thickness) (Figure 4a). Egg counts were manually performed in the uterus of adult parasites located within the renal tubules. A total of 457 eggs were counted, with a mean of 201.55 ± 100.12 eggs per parasite (n = 33) (Figure 4b).

## 4. Discussion

Renal trematodes affecting seabirds mainly belong to the genus *Renicola* and are commonly associated with the consumption of bivalves, mollusks, and fish [11]. Despite this well-recognized host–parasite relationship, reports of renal trematodiasis in free-ranging penguins remain scarce. This finding has been documented in African penguins (*Spheniscus demersus*) in South Africa [12], Magellanic penguins (*Spheniscus magellanicus*) in Brazil [13], and little penguins (*Eudyptula novaehollandiae*) and Fiordland crested penguins (*Eudyptes pachyrhynchus*) in New Zealand [10]. These studies indicate that lesion severity and distribution vary among penguin species. In *S. demersus*, *Renicola soleni* infection was associated with minimal gross and microscopic alterations [12]. In contrast, *S. magellanicus* exhibited microscopic lesions comparable to those observed in *S. humboldti* in the present study, including tubular dilation, squamous metaplasia, epithelial hyperplasia, fibrosis, and interstitial inflammation [13]. These findings suggest that trematode-associated renal pathology in penguins may present with variable severity, potentially influenced by parasite burden and host condition. Large numbers of renal trematodes have been reported to debilitate affected birds, contributing to mortality and potentially influencing stranding events [14]. In the present cases, up to two parasites were observed within a single renal tubule. Increased parasitic burden in animals with poor body condition may exacerbate tissue inflammation due to an imbalance between host immune response and parasite virulence [15,16]. A similar pattern has been described in Atlantic puffins (*Fratercula arctica*), in which severe trematode-associated nephropathy, ureteritis, and muscle and fat atrophy were reported in 96.3% of affected individuals [17]. Regarding squamous metaplasia, although this lesion has been documented in birds and is commonly associated with hypovitaminosis A—which may have been present in emaciated animals (Animal IDs 2, 3, and 4)—the severity and distribution of the lesions observed in this study support a direct association with trematode-induced inflammation [13]. Notably, the smooth muscle lesions observed in the ureters have not been previously reported in penguins and are likely related to the severity of the parasitic infection, potentially impairing ureteral function. Morphometric analyses demonstrated that adult parasites exhibited lengths comparable to those reported for other *Renicola* species, as did egg dimensions [10,11,13]. In the present study, parasite identification was based exclusively on histological sections. However, species-level identification using published taxonomic keys remains challenging due to the large number of eggs occupying the uterus of adult parasites, which limits the morphological assessment of key diagnostic structures. In this context, the incorporation of molecular approaches in future studies would be highly valuable to improve pathogen identification and taxonomic resolution, as well as to enable parasite isolation and a more comprehensive morphological characterization beyond tissue sections.

## 5. Conclusions

This study supports that stranding and mortality in Humboldt penguins are likely multifactorial processes, in which renal trematode infection may represent a contributing factor. The severity of renal and ureteral lesions observed suggests that trematodiasis should be considered within the context of emerging diseases affecting this species and may be relevant to conservation frameworks such as the national RECOGE plan. Morphological evaluation of adult parasites and eggs provides useful preliminary information for taxonomic assessment; however, definitive species identification requires molecular analyses, including PCR and sequencing. Such approaches are essential for accurate classification of these trematodes and for improving the understanding of their life cycle.

## Figures and Tables

**Table 1:** Data from Humboldt penguins with renal and ureteral lesions associated with trematodes stranded along the Chilean coast in 2025.

Animal ID	Stranding Report	Stranding Condition	Age Category	Sex	Body Score	Stranding Date	Date of Death	Geographic Location
1	13975	Dead	Adult	F	Under conditioned	15 July 2025	Unknow	29°54′14″ S, 71°16′27″ O
2	14182	Alive	Juvenile	M	Emaciated	19 September 2025	20 September 2025	29°52′58″ S, 71°16′22″ O
3	14165	Alive	Juvenile	F	Emaciated	13 September 2025	26 September 2025	27°30′32″ S, 70°53′12″ O
4	14167	Alive	Juvenile	M	Emaciated	28 October 2025	29 October 2025	29°56′40″ S, 71°17′28″ O
5	14507	Alive	Adult	M	Normal	26 November 2025	26 November 2025	29°18′21″ S, 71°21′52″ W

## Data Availability

The data presented in this study are available on request from the corresponding author due to legal reasons and privacy regulations.

## Supplementary Materials

The following supporting information can be downloaded at: https://www.mdpi.com/article/10.3390/ani16060859/s1, Table S1: Pathology; Table S2: Eggs.

## Abbreviations

The following abbreviations are used in this manuscript:

CITES	Convention on International Trade in Endangered Species of Wild Fauna and Flora
RECOGE	Recovery, Conservation, and Management Plan
